# Adaptation across consecutive night shifts at 71°N under Arctic summer daylight and winter darkness: Effects on alertness, sleepiness, and fatigue

**DOI:** 10.5271/sjweh.4295

**Published:** 2026-07-01

**Authors:** Andreas N Holme, Line Victoria Moen, Mikael Sallinen, Kristian Bernhard Nilsen, Charlotte N Boccara, Andrew JK Phillips, Fred Haugen, Dagfinn Matre

**Affiliations:** 1 National Institute of Occupational Health in Norway Oslo, Norway.; 2 Faculty of medicine, University of Oslo, Oslo, Norway.; 3 Finnish Institute of Occupational Health, Helsinki, Finland.; 4 Section for clinical neurophysiology, Department of Neurology, Oslo University Hospital, Norway.; 5 Institute of clinical medicine, University of Oslo, Oslo, Norway.; 6 Norwegian Centre for Molecular Biosciences and Medicine, University of Oslo, Oslo, Norway.; 7 Flinders Health and Medical Research Institute (Sleep Health), Flinders University, Bedford Park, Australia.

**Keywords:** circadian disruption, circadian rhythm, high latitude, light exposure, observational crossover study, photoperiod, polar day/night, psychomotor vigilance, seasonal variation, shift work

## Abstract

**Objectives:**

This study aimed to investigate how alertness, sleepiness, and fatigue change across consecutive night compared to morning shifts among Arctic shift workers and whether these effects differ between seasons of midnight sun and polar night.

**Methods:**

We conducted an observational crossover study of 118 shift workers from an industrial plant at a high latitude (71°N) in northern Norway. Eighty-one individuals participated in both the light (near 24-hour daylight) and dark (minimal natural light) seasons. Work schedules included blocks of seven consecutive morning shifts and seven consecutive night shifts, separated by four rest days. Alertness (psychomotor vigilance test), subjective sleepiness (Karolinska Sleepiness Scale), and subjective fatigue were measured at the end of shifts on days 1, 3, and 6 of each shift block. We analyzed data using multilevel mixed-effects regression models with season, shift type (morning/night), and consecutive workday number as fixed effects.

**Results:**

Night shifts were linked to lower alertness and higher sleepiness and fatigue in both seasons, with the largest impairments on the first night. Across six consecutive night shifts, alertness improved and sleepiness and fatigue decreased, with similar trajectories in both seasons. There was no evidence that season significantly affected alertness, sleepiness, or fatigue.

**Conclusions:**

Night shifts generally impair alertness and increase sleepiness and fatigue, yet outcomes improved across consecutive nights. Despite the well-established effects of natural light on circadian rhythms, the seasonal photoperiod altered neither the shift-related impairments in alertness, sleepiness or fatigue nor the subsequent improvements across consecutive nights; workers showed similar adaptation in both seasons.

Night shifts disrupt the circadian timing system by misaligning the sleep/wake cycle with external cycles such as the light/dark cycle, leading to circadian disruption (1) and sleep disturbances that are considered key drivers of the health risks associated with shift work (2). Night work is associated with increased sleepiness, reduced cognitive abilities, and higher accident risk (3, 4). Night work is also associated with fatigue, with mental fatigue being most relevant during safety-sensitive around-the-clock operations (5). Understanding these effects is crucial to more effectively mitigate health risks, reduce accidents, and improve job performance.

Light serves as the primary time cue for human circadian rhythms (6). It is well established that exposure to light before the circadian nadir (the lowest point of the endogenous core body temperature rhythm) causes a phase delay, whereas exposure after the nadir causes a phase advance of circadian rhythms (7). The regulation of daily changes in alertness and sleepiness is closely linked to circadian rhythms (8). Since light exposure affects the rate at which circadian rhythms entrain to a new schedule among shift workers (8, 9), seasonal changes in photoperiod are expected to influence their adaptation to night work. Most shift-work research focuses on settings with distinct daytime light and nighttime darkness; far less is known about how seasonal daylight patterns, including periods with little day-night light difference, influence outcomes, such as fatigue and cognitive function.

At high latitudes, such as the Arctic, the extreme daylight variations across seasons provide a unique context for studying circadian disruption. Phenomena such as the midnight sun (the sun remains above the horizon through the night) and polar night (the sun does not rise above the horizon) may differentially challenge mechanisms linked to circadian entrainment. Periods of midnight sun involve continuous nighttime solar irradiance of biologically relevant light (10, 11) that remains well above the physiological threshold required for significant circadian phase resetting and melatonin suppression (6, 12). For non-shift workers, the impact on circadian rhythms is highly behavioral; the continuous daylight may act as both a phase-delaying and phase-advancing cue, making the net effect dependent on individual routines and yielding mixed results in the literature (11, 13, 14). In contrast, shift workers cannot freely adjust their routines, and it remains unknown how these environmental extremes interact with night work to alter circadian adaptation and health. During night work in the light season, daylight typically overlaps both phase-advancing and phase-delaying time windows. During the dark season, the absence of natural light may weaken synchronization of circadian rhythms to local time. Short photoperiods have been associated with phase delays in Antarctica (13, 15), controlled studies (16) and real-world conditions (17). Because transitioning from morning to night shifts typically involves delaying circadian rhythms, it is expected that it will be easier for night workers to adapt during the dark season. The lack of morning light during the dark season is anticipated to further facilitate this phase delay as light exposure following night shifts typically inhibits circadian alignment, thereby exacerbating the negative effects of circadian disruption (18, 19).

Here we present the results of a longitudinal study investigating how seasonal variations interact with consecutive morning and night shifts to impact alertness, sleepiness and fatigue. We tested two hypotheses (H). H1: Compared with morning shifts, night shifts reduce alertness and increase sleepiness and fatigue; we will test whether the magnitude of these effects is moderated by season (near 24-hour daylight versus winter darkness). H2: Across consecutive night shifts, workers will show signs of adaptation, demonstrated by increased alertness and decreased sleepiness and mental fatigue, during the dark season, whereas they will show little or no such adaptation during the light season.

## Methods

### Participants

The study recruited 118 of 150 invited process operators from an industrial plant in northern Norway (71°N) (20). The cohort included a mix of workers whose primary shifts were located indoors, outdoors, or a mix of both. The plant's outdoor areas were illuminated with artificial lighting, but we did not quantify individual exposure to natural and artificial light, and characterizing this variability was beyond the aims of the present study. Three crews were recruited during the light season (N=61) and three during the dark season (N=51), including 81 who participated in both seasons. Participants were recruited by the research team through paper flyers and at startup information meetings. The Norwegian Regional Committee for Medical Research Ethics (REK) South-East D reviewed the project and determined that it falls outside the scope of the Norwegian Health Research Act (cf. § 2 and § 4 letter a; reference 495816).

### Study design and work schedule

**Figure 1:**
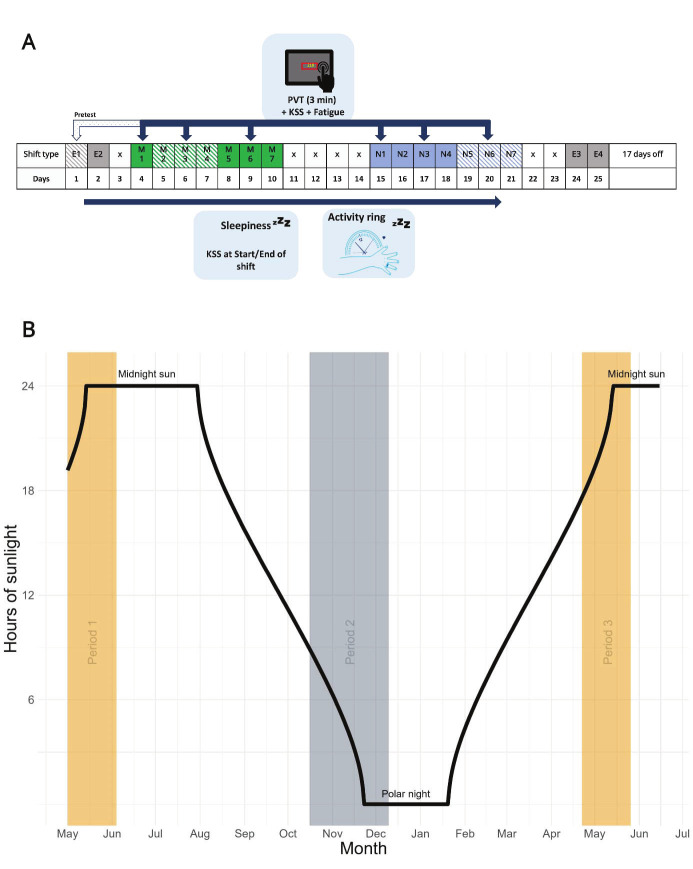
A) Overview of the study's shift schedule and data collection protocol. E: Evening shift (14:00–23:00), M: Morning shift (07:00–14:00), N: Night shift (23:00–07:00), X: Day off. Angled shading: 12-hour shift (07:00–19:00 or 19:00–07:00). Alertness, sleepiness and fatigue were measured between 12:30–15:00 on M1, M3, M6 and between 05:00–07:30 on N1, N3, N6, in addition to a pretest during E1. Sleepiness was also assessed daily at the start and end of each shift between E2 and N7, during which subjects also wore an activity ring measuring sleep. B) Hours of sunlight derived from sunrise and sunset timings, with periods of data collection highlighted in orange (light season) and grey (dark season).

Each participant was followed for two seasons in an observational crossover design. Workers followed a 25-day rotating shift schedule that included seven consecutive morning shifts and seven consecutive night shifts, with four days off in between (figure 1a). Morning shifts lasted 7 hours (07:00–14:00) and night shifts 8 hours (23:00–07:00), with 12-hour shifts on weekends (07:00–19:00 or 19:00–07:00). Data collection was conducted in three periods to accommodate the crossover design for two cohorts (i): May–June 2023 (“light season”); (ii) October–December 2023 (“dark season”); and (iii) April–May 2024 (“light season”). One cohort started in period 1 and finished in period 2, while the other started in period 2 and finished in period 3. These windows did not fully coincide with the absolute polar night or midnight sun because the plant operated a different shift roster later in summer. The selected periods preserved large contrasts in daily sunlight (figure 1b) while keeping mean temperatures comparable (dark: −1.9 °C; light: 4.1 °C), thereby reducing thermal confounding.

Alertness, sleepiness, and fatigue were assessed using a tablet (iPad 9^th^ generation) towards the end of morning shifts (M1, M3, M6), and night shifts (N1, N3, N6) in a 2.5-hour drop-in window for convenience (12:30–15:00 for morning shifts and 05:00–07:30 for night shifts; figure 1a). During the 12-hour M3 shift, testing followed the same fixed clock windows as M1 and M6 for all participants, ensuring consistent timing. M6 and N6 were chosen over M7/N7 due to practical considerations. Sleepiness was additionally assessed at the start and end of each of all work shifts (M1–7 and N1–7). To familiarize participants with the procedure and mitigate learning effects, a pretest comprising three consecutive alertness tests was conducted prior to the test days. The study is reported using the STROBE guidelines for cohort studies (the completed checklist is available in the supplementary material, URL).

### Measurements

We assessed alertness with a 3-minute version of the psychomotor vigilance test (PVT) (21), using software designed for iPad (Joggle Research, version 3.7; iPadOS 16.3.1). The PVT is a commonly employed tool to measure reaction time (RT) and detect cognitive impairments associated with circadian disruption (21, 22). While using a shorter PVT version may mask performance impairments that emerge during longer sessions, it remains a validated, sensitive tool that likely contributed to increased participant compliance (23). Response speed (1/RT) and lapses (RT>355 ms) were used as performance metrics (see supplementary methods S1 for more details).

Subjective fatigue was assessed on tablets prior to tests of alertness. Participants answered the question “*How mentally fatigued have you felt today?*” on a 0–10 numerical rating scale, anchored as 0=not at all and 10=to a very large extent.

Subjective sleepiness was assessed by the Karolinska Sleepiness Scale (KSS) (24), a numerical rating scale from 1 (very alert) to 9 (very sleepy, fighting sleep, effort to keep awake) (supplementary methods S3). The KSS has previously been used as a sensitive indicator of insufficient sleep and impaired cognitive function (25). The KSS was conducted at different points in the experimental protocol: (i) prior to alertness tests via a tablet, and (ii) daily at the start and end of shifts by completing an assessment on a sheet of paper (figure 1a).

Participants' age, sex, height, weight, and caffeine consumption were collected via an electronic baseline questionnaire, which also included questions about melatonin and sleep medicine use, as well as insomnia symptoms (supplementary methods S4). Chronotype was determined through a validated version of the Munich ChronoType Questionnaire for shift workers (MCTQ^Shift^) (26). Based on the corrected midpoint of sleep on free days (MSFsc), participants were classified as early (≤02:59), intermediate (03:00–04:59), or late (≥05:00) chronotypes. Sleep duration of periods ≥3 hours was estimated by a smart ring, Oura Gen3 (Oura Health Oy, Oulu, Finland), worn from E1 to after N6 (figure 1a). Total sleep duration and time awake (hours since sleep offset) were retrieved for the last sleep episode within 24 h prior to tablet tests. The effect of season on sleep will be analyzed in a separate article.

Employer payroll data provided objective working hours. By cross-referencing these data with the assigned work schedules, we identified deviations, such as participants working additional shifts or being assigned to a different shift type than originally scheduled. If a participant worked a night shift within two days prior to a scheduled block of seven consecutive morning or night shifts, any subsequent observations from that block were excluded from the analyses. Additionally, if participants worked an unscheduled shift or were absent during a block, all remaining observations for the corresponding block were also removed. This ensured that findings reflected consistent and intended exposure conditions.

### Sample size and power

Based on similar night shift studies measuring KSS, lapses, and 1/RT, we expected effect sizes of Cohen's d of 0.3–0.4, when comparing N1 and N3 (80% power, α=0.05, two-sided). This yields a sample size of 51–89.

### Statistical analysis

We selected linear mixed models (LMM) to analyze the multilevel structure and account for clustering within participants. Outcome variables included alertness (PVT response speed and lapses), sleepiness, and fatigue. Models were identical for all outcome variables, except lapses, for which a generalized linear mixed model (GLMM) was used due to its count data characteristics and potential overdispersion.

Models included fixed effects for shift type (morning versus night), season (light versus dark) and consecutive shifts (day 1, 3, 6), along with two- and three-way interactions. In exploratory analyses, we replaced the season variable with objective light exposure metrics: daily sunlight hours (from sunrise/sunset) and 24-hour incident solar irradiance (NASA POWER; supplementary methods S5), centered and scaled.

To account for potential confounding factors, we tested age, sex, chronotype, sequence (dark-first versus light-first) and hours since shift start as covariates in the models through forward selection. To maintain a uniform model structure, variables were retained only if they yielded a lower Akaike Information Criterion (AIC) and were selected for ≥2 of the outcome variables (response speed, lapses, sleepiness, and fatigue). In addition, we tested the inclusion of time awake and sleep duration as fixed effects in the models to explore the indirect effects of sleep behavior. “Participant” was added as a random effect and random intercept models were compared to random slope models. To address potential clustering within shift crews (eg, workload differences), crew was considered as an additional random effect. Model selection for fixed-effect structures was conducted using maximum likelihood (ML) and the lowest AIC; final LMM were refit using restricted maximum likelihood (REML) for parameter estimation. GLMM were fit by ML and missing data were assumed missing at random.

Models were summarized using estimated marginal means (EMM). To evaluate adaptation across shifts, we conducted pairwise comparisons among consecutive shifts within shift type, with Bonferroni adjusted P-values. Sleepiness measured at the beginning and the end of every shift allowed us to analyze the adaptation to night shifts with seven-point temporal resolution, treating consecutive shifts (1–7) as a continuous variable to simplify the model. For this model, only KSS data recorded at the *end of night shifts* were used to prevent confounding effects from the differing start and end times of the 12-hour shifts.

Data processing and statistical analyses were conducted in R (version 4.4.1), running on Windows 11. The packages 'lme4' (version 1.1-35.5) and 'glmmTMB' (version 1.1.10) were employed for LMM and GLMM fitting. Tests were conducted using 'lmerTest' and EMM were calculated using the 'emmeans' package (version 1.11.1). All tests were set to a 5% significance level.

### Final models

The final models included shift type, consecutive shifts, season, sex, and age as fixed effects. In exploratory models replacing season (light/dark) with objective light exposure metrics (daily sunlight hours; 24-hour solar irradiance), model fit did not improve, so season was retained as the reporting factor. We included participant as both a random intercept and random slopes for shift type (morning/night).

In contrast to alertness and fatigue models, adding sleep duration and time awake improved the fit of the KSS model. However, the other fixed-effect estimates were largely unchanged. To maintain consistency across outcomes and avoid reducing the dataset due to missing sleep data, we excluded sleep variables from all final models.

## Results

Out of 118 recruited participants, 112 (89 men, 23 women) contributed to the data collection, with 81 participating in both seasons. Six participants were excluded due to early withdrawal or incompatible work schedules (supplementary figure S1). Participant characteristics are presented in table 1.

**Table 1:** Baseline characteristics of study participants by season of participation. Values are from the baseline questionnaire, with participants grouped into three categories: those who participated in both light and dark seasons (both), only in the dark (only dark), or only in the light (only light). [SD=standard deviation; NA=not available.

Variable		Season								P-value ^a^
		Both (N=81)			Only dark (N=18)			Only light (N=13)		
		Mean (SD)	N (%)		Mean (SD)	N (%)		Mean (SD)	N (%)	
Age		30.98 (10.75)			32.78 (10.84)			37.77 (13.68)		0.14
Body mass index		26.78 (4.14)			27.09 (4.71)			26.29 (5.26)		0.9
Caffeine (daily units)		2.95 (1.99)			2.65 (2.18)			3.54 (1.98)		0.3
	NA ^b^	0			1			0		
Sex										0.8
	Female		18 (22)			3 (17)			2 (15)	
	Male		63 (78)			15 (83)			11 (85)	
Chronotype ^c^										0.080
	Early		8 (9.9)			1 (8.3)			3 (30)	
	Intermediate		34 (42)			7 (58)			6 (60)	
	Late		39 (48)			4 (33)			1 (10)	
	NA ^b^		0			6			3	
Melatonin ^d^			20 (25)			2 (12)			3 (23)	0.6
	NA ^2^		1			1			0	
Sleep medicine ^d^			1 (1.4)			0 (0)			0 (0)	>0.9
	NA ^b^		8			1			0	
Insomnia ^e^			44 (54)			9 (53)			5 (38)	0.6
	NA ^b^		0			1			0	

^a^ Kruskal–Wallis tests (continuous); Fisher’s exact or Pearson’s Chi-squared (categorical). ^b^ NA rows show the number of missing responses for each item within each season group. ^c^ Classified by the midpoint of sleep on free days corrected for sleep debt (MSFsc) derived from MCTQShift (early: ≤02:59; intermediate: 03:00–04:59; late: ≥05:00). Due to incomplete data, 46 participants were classified based on midpoint of sleep on free days (MSF), rather than MSFsc. ^d^ Number of participants reporting melatonin or sleep medicine use (supplementary methods S4). ^e^ Insomnia classified using Bergen Insomnia Scale–based criteria (supplementary methods S4).

The random effects structure of the models indicated large variability across participants (see supplementary table S1).

### Alertness

**Figure 2:**
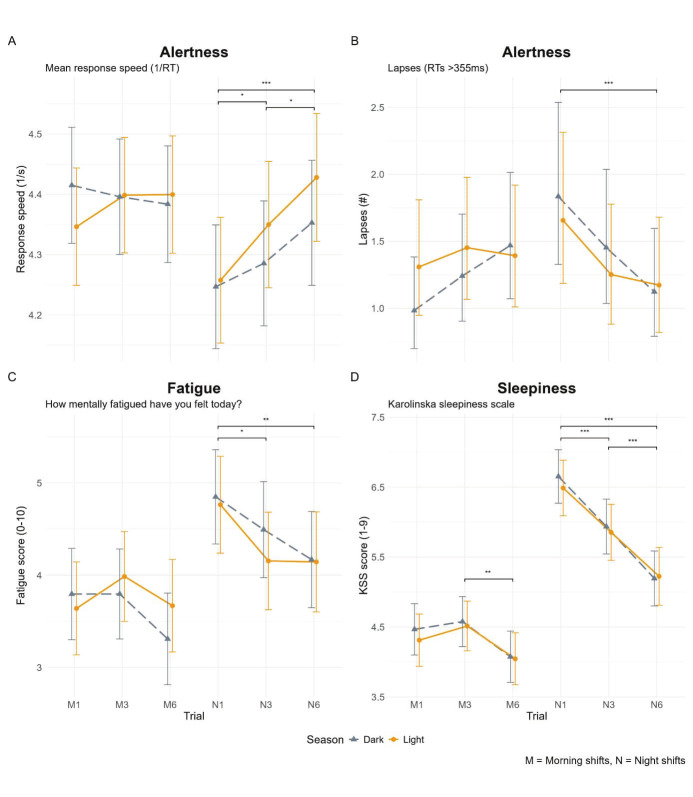
Estimated marginal means (EMM) of alertness, sleepiness, and fatigue across consecutive morning (M1,3,6) and night (N1,3,6) shifts show significant differences between consecutive shifts, but no significant seasonal effect. Panel A displays alertness as measured by the psychomotor vigilance test (PVT), quantified through response speed (inverse response time). Panel B presents alertness assessed by lapse counts (omissions >355ms). Sleepiness, from the Karolinska Sleepiness Scale (KSS), is shown in panel C. Subjective fatigue, assessed on a scale from 0–10, is illustrated in panel D. EMM for the light season are represented by filled circles connected by a solid line, while those for the dark season are depicted as triangles and dashed lines. Each data point is accompanied by 95% confidence intervals.  Significance bars represent pairwise comparisons of estimated marginal means stratified by morning/night condition. Results are averaged across season and sex. P-values are Bonferroni-adjusted and indicated by *P<0.05; **P<0.01; ***P<0.001. Only parts of the y-axis scale are shown for visibility and to highlight data differences.

*Response speed.* Night shifts were associated with a significantly lower mean response speed compared to morning shifts (EMM 4.32 versus 4.39 1/s; P<0.001; table 2). While there was no main effect of consecutive shifts, a significant interaction between the first and the sixth consecutive night shift was observed (P=0.007; table 2). Specifically, response speed was at its lowest during the first night shift (N1) and improved over subsequent night shifts, but not over consecutive morning shifts. Pairwise comparisons of estimated marginal means (EMM) for consecutive shifts, stratified by shift type, showed significant differences from N1 (EMM 4.25 1/s) to N3 (EMM 4.32 1/s; P=0.032), and further from N3 to N6 (EMM 4.39 1/s; *P=0*.017; N1 versus N6 P<0.001; figure 2a). Notably, the changes in response speed across consecutive shifts were consistent across all six shift crews, for both seasons (supplementary figure S2).

**Table 2:** Model summary for alertness (PVT response speed and lapses), sleepiness (KSS) and fatigue. Fixed effects and interactions for all model outcomes are shown. Note that lapse estimates are shown as incidence rate ratios. N refers to the total number of test sessions. [CI=Confidence Interval; KSS=Karolinska Sleepiness Scale; M=Morning shift; N=Night shift].

Predictor		Response Speed					Lapses				KSS				Fatigue		
		N	Estimate	95% CI	P-value		Incidence rate ratio	95% CI	P-value		Estimate	95% CI	P-value		Estimate	95% CI	P-value
(Intercept)		926	4.3 ***	4.1–4.5	<0.001		1.38	0.70–2.69	0.4		5.1 ***	4.4–5.7	<0.001		4.4 ***	3.4–5.5	<0.001
Shift type																	
	M	478															
	N	448	-0.17 ***	-0.24– -0.09	<0.001		1.87 ***	1.31–2.66	<0.001		2.2 ***	1.7–2.7	<0.001		1.1 ***	0.53–1.6	<0.001
Consecutive day																	
	1	309															
	3	319	-0.02	-0.09–0.05	0.6		1.26	0.90–1.77	0.2		0.11	-0.31–0.53	0.6		0.00	-0.49–0.49	>0.9
	6	298	-0.03	-0.10–0.04	0.4		1.49 *	1.07–2.09	0.020		-0.39	-0.82–0.04	0.074		-0.49	-0.99–0.02	0.058
Season																	
	Dark	476															
	Light	450	-0.07	-0.14–0.00	0.066		1.33	0.93–1.90	0.11		-0.16	-0.60–0.29	0.5		-0.16	-0.67–0.36	0.6
Sex																	
	Female	193															
	Male	733	0.23 **	0.06–0.41	0.009		0.43 ***	0.27–0.70	<0.001		-0.18	-0.63–0.26	0.4		0.15	-0.59–0.89	0.7
Age		926	0.00	-0.01–0.01	>0.9		1.00	0.99–1.02	0.8		-0.02	-0.03–0.00	0.051		-0.02	-0.05–0.00	0.094
Shift type * Consecutive day		926															
	N * 3	149	0.06	-0.04–0.16	0.2		0.63 *	0.40–0.98	0.042		-0.83 **	-1.4– -0.23	0.007		-0.36	-1.1–0.34	0.3
	N * 6	142	0.14 **	0.04–0.24	0.007		0.41 ***	0.26–0.65	<0.001		-1.1 ***	-1.7– -0.46	<0.001		-0.19	-0.90–0.51	0.6
	Shift type * Season	926															
	N * Light	212	0.08	-0.02–0.18	0.13		0.68	0.43–1.08	0.10		-0.01	-0.63–0.61	>0.9		0.07	-0.65–0.79	0.8
Consecutive day * Season		926															
	3 * Light	158	0.07	-0.03–0.17	0.2		0.88	0.55–1.39	0.6		0.09	-0.50–0.69	0.8		0.35	-0.35–1.0	0.3
	6 * Light	143	0.08	-0.02–0.19	0.10		0.71	0.45–1.14	0.2		0.13	-0.48–0.74	0.7		0.52	-0.20–1.2	0.2
Shift type * Consecutive day * Season		926															
	N * 3 * Light	72	-0.02	-0.16–0.12	0.8		1.09	0.58–2.04	0.8		-0.01	-0.86–0.84	>0.9		-0.60	-1.6–0.39	0.2
	N * 6 * Light	66	-0.02	-0.16–0.12	0.8		1.62	0.85–3.11	0.14		0.07	-0.80–0.94	0.9		-0.46	-1.5–0.56	0.4

* P<0.05. ** P<0.01. *** P<0.001.

The analysis did not reveal any main effects or interaction effects of season on response speed. Additionally, male participants had significantly higher response speed compared to females (EMM 4.47 versus 4.24 1/s; P=0.009), while age did not influence response speed (table 2).

*Lapses.* Night shifts significantly increased the number of lapses compared to morning shifts [incidence rate ratio (IRR) 1.87, P<0.001; table 2]. However, the overall number of lapses was low (mean 1.8, standard error=0.1, N=926), and 41% of test runs showed no lapses (supplementary table S2). A significant main effect of the sixth consecutive shift indicated an increase in lapses compared to the first morning shift (IRR 1.49, P=0.02). A significant interaction between shift type and consecutive shifts revealed that the highest number of lapses occurred on N1, with a decrease across N3 (IRR 0.63, P=0.042) and N6 (IRR 0.41, P<0.001). Furthermore, pairwise comparisons of EMM confirmed a significant difference between N1 and N6 (P<0.001), no significant difference between morning shifts (all P>0.15), while the contrast between N1 and N3 showed only a trend (P=0.062; figure 2b).

No seasonal effects on lapses were detected. Additionally, male participants exhibited fewer lapses compared to females (IRR 0.43, P<0.001), with no effect of age on lapse rates.

### Fatigue

Subjective fatigue was significantly higher at the end of night compared to morning shifts (EMM 4.43 versus 3.70; P<0.001; table 2). None of the other variables in the model were significantly associated with fatigue. However, contrast analysis of EMM revealed that N1 (4.81) was associated with the highest fatigue scores, with a significant reduction on N3 (4.32; P=0.024) and N6 (4.16; P=0.001; figure 2c).

### Sleepiness

*Sleepiness prior to PVT tests.* Night shifts were associated with higher levels of sleepiness compared to morning shifts (EMM 5.89 versus 4.33; P<0.001; table 2). There was a significant interaction between shift type and consecutive shifts: sleepiness peaked on N1 (EMM 6.57) and decreased on N3 (EMM 5.90) and further on N6 (EMM 5.21). Pairwise comparisons of EMM showed significant differences among all three night shift measurements (all P<0.001; figure 2d). There was no effect of season or any interaction effects between shift type and season, on sleepiness (table 2). The changes in sleepiness across consecutive shifts were consistent across all six shift crews, for both seasons (supplementary figure S2).

**Figure 3:**
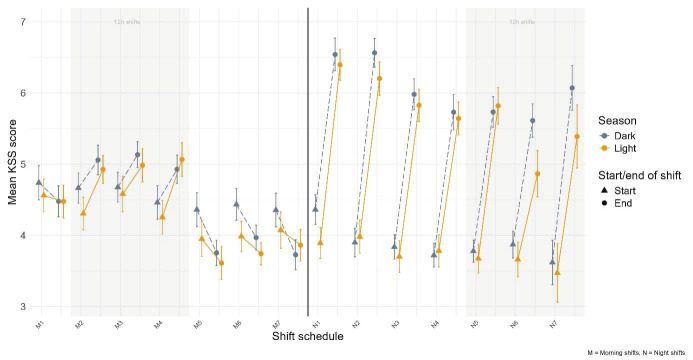
Sleepiness across consecutive morning (M1–7) and night shifts (N1–7). Filled triangles denote the start of each shift, connected by lines to circles that represent the end of the shift. These symbols illustrate the overall mean scores, accompanied by error bars displaying standard error. Long-lasting shifts (12 h) are highlighted by a grey background. The full KSS scale is 1–9, while only a part is shown in the figure. The number of responses for each sampling point ranged from 37–76, with a notable decline during N7 (range: 17–34).

*Sleepiness at start and end of shifts.* The model for sleepiness at the start and end of each shift showed a small but statistically significant main effect of season, with lower KSS scores in the light season (mean difference: 0.18, P=0.002; supplementary table S3). Scores varied by type of shift (morning versus night), the timing within the shift (start versus end) and shift length (8 versus 12-hour morning shifts) (figure 3; supplementary table S3). Notably, the highest sleepiness levels were observed at the end of night shifts.

The subset analysis of end-of-night-shift data showed a significant main effect of consecutive night shifts, with sleepiness scores declining across successive shifts (P<0.001; supplementary table S3). No seasonal or other variable effects were detected in this model.

## Discussion

We examined how seasonal differences (near 24-hour daylight versus winter darkness) interact with consecutive morning and night shifts to influence alertness, sleepiness, and mental fatigue. We found that although night shifts impaired alertness and elevated sleepiness and fatigue in both seasons, these outcomes progressively improved over consecutive nights. Ultimately, seasonal photoperiod did not modify these patterns; workers appeared to adapt regardless of the extreme differences in natural light.

We found support for parts of our first hypothesis that night shifts negatively impact alertness, sleepiness, and fatigue. However, this effect did not seem impacted by seasons. Reduced performance during night shifts aligns with prior research showing that individuals adapted to a daytime schedule experience increased sleep pressure and impaired cognitive performance near their circadian nadir (4, 27). The daily sleepiness assessments at the start and end of shifts (figure 3) also follow within-shift patterns seen in previous studies of similar shift schedules (25). These scores were slightly elevated during the dark season; however, this seasonal effect was not evident in the subset analysis of end-of-night shifts, nor in the sleepiness recorded prior to the PVT tests. This discrepancy was likely driven by a seasonal effect on sleepiness at the start of morning shifts, possibly due to the immediate alerting effect of the high-intensity morning daylight during the light season.

Contrary to our second hypothesis, seasonal differences did not significantly influence adaptation rates to night work in our experimental setting. Across seasons, impairments were greatest on the first night shift, then sleepiness and mental fatigue declined and alertness improved across subsequent shifts, approaching morning-shift levels by the sixth night. This season-independent behavioral pattern aligns with high-latitude studies showing similar season-independent phase delays in circadian markers under delay-favoring schedules (28, 29), though seasonal variation can occur under phase-advancing regimens (30). Because we did not follow participants after the work block ended, their readaptation to a daytime schedule remains unknown, and seasonal influences may be more pronounced during this phase (29).

As our schedule favored a phase delay, we anticipated that the absence of morning daylight during the dark season would facilitate adaptation to night shifts, whereas morning daylight in the light season could counteract adaptation by promoting a phase advance. At the same time, light exposure before and at the start of night shifts in the light season may suppress melatonin and support delay. This interplay of phase-advancing and phase-delaying influences creates a complex, hard-to-predict net effect that may explain the lack of seasonal differences in our data. We further speculate that the observed adaptation across night shifts, a pattern infrequently reported in the literature, may reflect small day-night differences in natural light during both seasons (10). Because a robust day-night contrast is essential for tightly entraining the circadian pacemaker to the 24-hour solar day, diminishing this contrast reduces the strength of the light-dark cycle as a zeitgeber (31). This weakened environmental entrainment may in turn make the circadian system more susceptible to behavioral time cues (such as scheduled work, meal times and sleep) and artificial lighting, thereby facilitating rapid phase shifts and adaptation to night work in both seasons. These possibilities warrant targeted study using personal light measurements and sampling of transitional seasons with greater day-night contrast.

High interindividual variability in circadian traits, such as responsiveness to light (12, 32, 33), could further obscure seasonal effects if subgroups with differing phase responses are not adequately captured in the model parameters. Other contributing factors may be limited outdoor exposure and increased use of artificial light, both of which reduce sensitivity to seasonal cues (34). The circadian system is highly sensitive to light, and even low intensities, such as typical room light, may suppress melatonin production (12). Participants' sleep/wake schedules were constrained by their work schedule, and seasonal effects on circadian rhythms might have been more pronounced with more flexible sleep timing.

Our finding that PVT performance improved across consecutive night shifts aligns with laboratory studies of simulated shift work (35), but contrasts with many real-world field studies. In typical field settings shift workers rarely adapt across consecutive night shifts; studies commonly report impairments in PVT performance (19, 36), or no change at all (27). The discrepancy between our findings and these field studies may stem from the unique environmental context of our Arctic cohort. PVT performance fluctuates across the 24-hour cycle due to the combined influence of circadian rhythms and time awake, which are difficult to disentangle (37). While extended wakefulness before the first shift intuitively explains the severe initial performance drop, adding sleep duration and time awake as covariates in our models did not improve model fit, suggesting that improvements in alertness across night shifts are at least partly attributable to changes in circadian rhythms. Most night workers struggle to shift their underlying biological rhythms (38), and adaptation is often limited to specific subgroups (33). However, the high-latitude light environment at 71°N, characterized by small day-night differences in natural light during both seasons, may help explain why we observed behavioral indicators of adaptation that are rarely reported in lower-latitude field studies.

Notable exceptions exist in specific work-settings that facilitate greater night shift adaptation. For instance, oil rig workers have successfully delayed their 6-sulfatoxymelatonin (aMT6s) rhythms in alignment with nocturnal schedules (28, 39) and improved alertness and sleepiness (40, 41). In our study, the increased alertness and reduced sleepiness and fatigue across six consecutive night shifts mirror these behavioral improvements, which in the oil rig cohorts were driven by circadian adaptation; it is tempting to speculate that a similar underlying biological shift may have occurred among our participants. Moreover, the patterns of sleepiness across night shifts in our study are similar to those of oil rig workers showing adaptation (40), albeit less pronounced. The adaptation observed in these oil rig studies has been explained by the limited daylight exposure post night shifts due to workers not facing commuting challenges after shifts, which may parallel the experience of workers in our study during the dark season. However, a critical distinction is that workers in our investigation are not isolated from their home environments and may retain commitments that hinder optimal adjustment to night shifts. Although we did not systematically collect residency data, most participants were local residents.

Although our real-world study likely included variation in external factors such as diet and lifestyle, we ensured comparable work conditions through strict exclusion criteria and similar tasks across shifts, a rare achievement in field studies. Moreover, using payroll data allowed precise tracking of shift timing, enhancing the reliability of the findings. Due to regulatory constraints, light exposure was not directly measured, limiting our ability to assess differences in intensity, timing, and composition, as well as the potential influence of artificial light. However, the use of two extreme seasons with vastly different daylight hours offered an ecologically valid framework for examining natural photoperiod effects. The relatively homogeneous population, primarily consisting of young males, may reduce generalizability of findings. Previous studies indicate that young age, male sex, and late chronotype, characteristics prevalent among our participants, are associated with higher shift work tolerance (42), which may partly account for the observed improvements across consecutive night shifts. A 'healthy worker effect' might have influenced the results, as individuals struggling to adjust to shift work are less likely to remain in the workforce. Nonetheless, our relatively large sample size and repeated measurements enhance the reliability of the findings by minimizing interindividual variability and supporting robust within-subject comparisons.

The mean sleepiness scores during the first night shift approached levels linked to performance impairments and elevated microsleep risk (25). While alertness changes across night shifts were more modest, they aligned with sleepiness and fatigue patterns, reinforcing our conclusions. Furthermore, even moderate decreases in cognitive performance observed in sleep-deprived individuals are comparable to those seen in individuals with alcohol levels exceeding legal driving limits (43). It must be noted that the morning shifts in our study involve very early waking times, which may cause circadian disruption, thereby impairing scores and reducing differences between shift types (44).

This study demonstrates that, while working night shifts impairs alertness and increases sleepiness and fatigue, shift workers show signs of adaptation across six consecutive shifts, during both dark and light seasons. Contrary to our hypothesis, seasonal variations in daylight did not significantly influence this adaptation. Conducted in a unique location with extreme seasonal variations in natural light, our findings provide valuable insights into real-world shift work adaptation, informing strategies for worker well-being and performance under challenging conditions.

## Supplementary material

Supplementary materials
