# Supplementary material for: Adaptation across consecutive night shifts at 71°N under Arctic summer daylight and winter darkness: Effects on alertness, sleepiness, and fatigue
Source: Scand J Work Environ Health. 2026 Jun 26;52(4):403–14. doi: 10.5271/sjweh.4295 (PMC13345117; doi:10.5271/sjweh.4295)
Supplement: Supplementary materials [file SJWEH-52-403-S001.pdf]

Adaptation across consecutive night shifts at 71°N under Arctic summer daylight and winter darkness: Effects on alertness, sleepiness, and fatigue[1]

by Andreas N Holme,[2] Line Victoria Moen, Mikael Sallinen, Kristian Bernhard Nilsen, Charlotte N Boccara, Andrew JK Phillips, Fred Haugen, Dagfinn Matre

1. Supplementary materials
2. Correspondence to: Andreas N Holme, National Institute of Occupational Health, Oslo, Norway. [E-mail: andreas.holme@stami.no]

Contents

Supplementary Methods S1: Alertness

Supplementary Methods S2: Fatigue

Supplementary Methods S3: Sleepiness

Supplementary Methods S4: Questions from baseline questionnaire

Supplementary Methods S5: Objective light exposure metrics

Supplementary Table S1

Supplementary Table S2

Supplementary Table S3

Supplementary Figure S1

Supplementary Figure S2

Supplementary References

Supplementary Methods S1: Alertness

PVT was performed on a tablet lying flat on a table. Participants were instructed to hover their finger over the screen and response time (RT) was measured from the presentation of a stimulus to the finger touching the screen, a method comparable to the physical button approach (Kay et al., 2013). Stimuli consisted of a millisecond timer within a red box and included 1 second of feedback on response time after each trial, with an interstimulus interval of 2-5 seconds.

Supplementary Methods S2: **Fatigue**

The question used was slightly modified from the validated version used by Van Hooff et al. (2007) by adding the word "mentally" to the question. It is a potential limitation that this addition has not been validated, although it has been used before (Merkus, et al., 2015).

Supplementary Methods S3: **Sleepiness**

Karolinska Sleepiness Scale (KSS) (Åkerstedt & Gillberg, 1990), a 9-point numerical rating scale of self-reported scores verbally anchored as 1 = "Very alert", 3 = Alert, 5 = "Neither alert nor sleepy", 7 = "Sleepy, but no problem staying awake", and 9 = "Very sleepy, fighting sleep, effort to keep awake".

Supplementary Methods S4: Questions from baseline questionnaire

The questions below have been translated into English from the original questionnaire used in the study which was written in Norwegian.

Caffeine: *"Do you drink coffee or caffeinated beverages during the working day?",*

"How many units of coffee/caffeinated beverages do you consume during a workday/shift? Count a unit as a cup of coffee or a can/bottle of caffeinated beverage"

Sleep medicine: *"Do you use any of the following medications? Sleeping pills"*

Melatonin: *"Do you use any of the following medications? Melatonin"*

Insomnia*: *"How many times per week during the last month...*

1. *... Have you taken more than 30 minutes to fall asleep after the lights went out?"*
2. *... Have you been awake for more than 30 minutes in between sleep?"*
3. *... Have you woken up more than 30 minutes earlier than you would have liked, without being able to sleep again?"*
4. *... Have you felt too little rested after sleeping?"*
5. *... Have you been so sleepy/tired that it has affected school/work or private life?"*
6. *... Have you been dissatisfied with your sleep?"*

*Insomnia was classified using questions adapted from the Bergen Insomnia Scale (Pallesen et al., 2008), based on the following criteria: Insomnia (scores of 3 or more on one of the questions 1-4 and scores of 3 or more on one of questions 5-6) vs. non-insomnia (scores of 2 or lower on all questions 1-4 or scores of 2 or lower on all questions 5-6).

Supplementary Methods S5: Objective light exposure metrics

We quantified ambient solar energy at the study location for the 24-hour window preceding each test session using data from National Aeronautics and Space Administration (NASA) Langley Research Center's Prediction Of Worldwide Energy Resources (POWER) project funded through the NASA Earth Science Division. Specifically, we extracted the All Sky Surface Shortwave Downward Irradiance (ALLSKY_SFC_SW_DWN), which represents the horizontal irradiance (direct plus diffuse) incident at the surface under all-sky conditions. Hourly values were summed over the prior 24 hours to yield incident energy (kWh/m^2), time-aligned to the local test timestamp. Data were retrieved using the "nasapower" package in R (Sparks, 2018).

Supplementary Table S1

Supplementary Table S1: Random effects for the linear mixed models presented in Table 2.

Random Effects	**Response speed (1/s)**	**Lapses**	**KSS**	**Fatigue**
σ^2	0.05	0.62	1.82	2.48
τ_{00}	0.12 $_{\text{Id}}$	0.76 $_{\text{Id}}$	0.68 $_{\text{Id}}$	2.02 $_{\text{Id}}$
τ_{11}	0.01 $_{\text{Id.Day\_nightN}}$	0.12 $_{\text{Id.Day\_nightN}}$	0.91 $_{\text{Id.Day\_nightN}}$	0.68 $_{\text{Id.Day\_nightN}}$
ρ_{01}	0.46 $_{\text{Id}}$	0.40 $_{\text{Id}}$	-0.35 $_{\text{Id}}$	-0.09 $_{\text{Id}}$
ICC	0.74	0.60	0.32	0.48
N	112 $_{\text{Id}}$	112 $_{\text{Id}}$	112 $_{\text{Id}}$	112 $_{\text{Id}}$
Observations	926	926	926	926
Marginal R^2 / Conditional R^2	0.062 / 0.752	0.081 / 0.634	0.240 / 0.483	0.053 / 0.504

Supplementary Table S2

Supplementary Table S2: Alertness, fatigue, and sleepiness scores, organized by season, shift type (morning/night), and number of consecutive shifts. Season is categorized as Dark or Light. "M/N" indicates Morning or Night shifts, while the numbers 1, 3, or 6 specify consecutive shift count. Measures of alertness from the Psychomotor Vigilance Task (PVT) include Response Speed (1/s) and Lapses (omissions >355ms). KSS (Karolinska Sleepiness Scale) evaluates sleepiness; and subjective fatigue scores range from 0 to 10. Response speed are shown as mean scores, while Lapses, Fatigue, and KSS are provided as stratified counts and percentages within their respective score categories.

**Characteristic**	**Dark, M, 1** N = 77[1]	**Dark, M, 3** N = 84[1]	**Dark, M, 6** N = 79[1]	**Dark, N, 1** N = 83[1]	**Dark, N, 3** N = 77[1]	**Dark, N, 6** N = 76[1]	**Light, M, 1** N = 75[1]	**Light, M, 3** N = 86[1]	**Light, M, 6** N = 77[1]	**Light, N, 1** N = 74[1]	**Light, N, 3** N = 72[1]	**Light, N, 6** N = 66[1]
Response Speed (1/s)	4.48 (3.59, 5.56)	4.45 (3.31, 5.62)	4.42 (3.66, 5.47)	4.28 (3.27, 5.32)	4.32 (2.98, 5.05)	4.39 (3.26, 5.45)	4.42 (3.12, 5.24)	4.47 (3.25, 5.36)	4.48 (3.20, 5.49)	4.33 (2.86, 5.35)	4.42 (3.06, 5.33)	4.49 (3.30, 5.54)
Lapses												
0	37 (48%)	35 (42%)	30 (38%)	32 (39%)	31 (40%)	33 (43%)	27 (36%)	33 (38%)	30 (39%)	29 (39%)	29 (40%)	30 (45%)
1-2	27 (35%)	34 (40%)	29 (37%)	26 (31%)	30 (39%)	29 (38%)	35 (47%)	32 (37%)	29 (38%)	24 (32%)	31 (43%)	24 (36%)
3-5	12 (16%)	8 (9.5%)	14 (18%)	13 (16%)	9 (12%)	8 (11%)	9 (12%)	15 (17%)	11 (14%)	14 (19%)	6 (8.3%)	5 (7.6%)
6-8	1 (1.3%)	4 (4.8%)	3 (3.8%)	7 (8.4%)	2 (2.6%)	3 (3.9%)	2 (2.7%)	4 (4.7%)	3 (3.9%)	5 (6.8%)	3 (4.2%)	5 (7.6%)
>8	0 (0%)	3 (3.6%)	3 (3.8%)	5 (6.0%)	5 (6.5%)	3 (3.9%)	2 (2.7%)	2 (2.3%)	4 (5.2%)	2 (2.7%)	3 (4.2%)	2 (3.0%)
Fatigue												
0-1	14 (18%)	11 (13%)	14 (18%)	7 (8.4%)	13 (17%)	10 (13%)	19 (25%)	14 (16%)	12 (16%)	3 (4.1%)	7 (9.7%)	8 (12%)
2-3	38 (49%)	39 (46%)	37 (47%)	26 (31%)	25 (32%)	27 (36%)	29 (39%)	36 (42%)	32 (42%)	30 (41%)	29 (40%)	30 (45%)
4-5	16 (21%)	21 (25%)	23 (29%)	24 (29%)	24 (31%)	25 (33%)	19 (25%)	18 (21%)	25 (32%)	21 (28%)	28 (39%)	19 (29%)
6-7	8 (10%)	13 (15%)	5 (6.3%)	15 (18%)	13 (17%)	11 (14%)	6 (8.0%)	15 (17%)	6 (7.8%)	15 (20%)	6 (8.3%)	9 (14%)
8-10	1 (1.3%)	0 (0%)	0 (0%)	11 (13%)	2 (2.6%)	3 (3.9%)	2 (2.7%)	3 (3.5%)	2 (2.6%)	5 (6.8%)	2 (2.8%)	0 (0%)
KSS												
1-2	7 (9.1%)	3 (3.6%)	4 (5.1%)	1 (1.2%)	3 (3.9%)	2 (2.6%)	11 (15%)	7 (8.1%)	9 (12%)	1 (1.4%)	0 (0%)	5 (7.6%)
3-4	34 (44%)	41 (49%)	47 (59%)	10 (12%)	11 (14%)	23 (30%)	30 (40%)	41 (48%)	45 (58%)	10 (14%)	15 (21%)	18 (27%)
5-7	33 (43%)	37 (44%)	26 (33%)	46 (55%)	57 (74%)	46 (61%)	33 (44%)	34 (40%)	22 (29%)	43 (58%)	50 (69%)	40 (61%)
8-9	3 (3.9%)	3 (3.6%)	2 (2.5%)	26 (31%)	6 (7.8%)	5 (6.6%)	1 (1.3%)	4 (4.7%)	1 (1.3%)	20 (27%)	7 (9.7%)	3 (4.5%)

[1] Mean (Min, Max); n (%)

Supplementary Table S3

Supplementary Table S3: Model predictions of sleepiness scores (KSS): fixed effects from linear mixed models analyzing KSS scores assessed at the start/end of each shift M1-M7 and N1-N7 (left) and exclusively end of night shifts (right).

	**Full dataset**			**End of night shifts**		
Predictors	*Estimates*	*CI*	*p*	*Estimates*	*CI*	*p*
(Intercept)	5.22	4.59 – 5.84	**<0.001**	7.34	6.45 – 8.23	**<0.001**
Consecutive shift	-0.09	-0.15 – -0.04	**0.001**	-0.26	-0.37 – -0.14	**<0.001**
Start/End [Start]	0.27	-0.07 – 0.61	0.125			
Shift type [N]	2.09	1.70 – 2.48	**<0.001**			
Season [Light]	-0.18	-0.29 – -0.06	**0.002**	-0.16	-0.60 – 0.29	0.494
Sex [male]	-0.17	-0.60 – 0.25	0.421	-0.15	-0.79 – 0.49	0.645
Time of entry (12h shift) [Day]	0.85	0.64 – 1.06	**<0.001**			
Time of entry (12h shift) [Night]	0.20	-0.22 – 0.63	0.352			
Age	-0.01	-0.03 – 0.00	0.057	-0.01	-0.03 – 0.01	0.337
Consecutive shift × Start/End [Start]	0.02	-0.05 – 0.09	0.624			
Consecutive shift × Shift type [N]	-0.08	-0.15 – 0.00	0.062			
Start/End [Start] × Shift type [N]	-2.70	-3.21 – -2.19	**<0.001**			
(Consecutive shift × Start/End [Start]) × Shift type [N]	0.04	-0.10 – 0.19	0.539			
12h shift [yes]				0.30	-0.08 – 0.68	0.120
Consecutive shift × Season [Light]				-0.00	-0.11 – 0.11	0.998
**Random Effects**						
σ^2	2.06			1.63		
τ_{00}	0.78 $_{Id}$			1.37 $_{Id}$		
τ_{11}	0.44 $_{Id.Shift\_typeN}$			0.03 $_{Id.Consecutive\_shift}$		
ρ_{01}	-0.32 $_{Id}$			-0.29 $_{Id}$		
ICC	0.28			0.46		
N	111 $_{Id}$			97 $_{Id}$		
Observations	3251			695		

Supplementary Figure S1

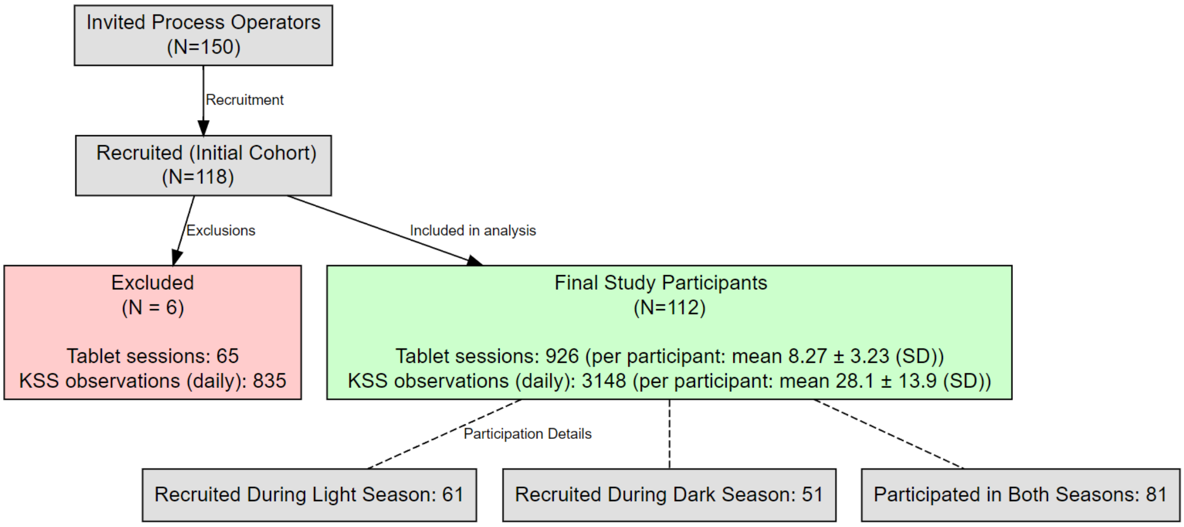

Supplementary Figure S1: Participant flow and data contributions. Of 150 invited process operators, 118 were recruited; 6 were excluded, yielding 112 final study participants included in the analysis. Tablet sessions denotes one completed test battery (KSS+Fatigue+PVT) while KSS observations daily refer to KSS scores assessed at the start/end of each shift M1-M7 and N1-N7.

Exclusions were based on early withdrawal or incompatible working hours (see main text for specific exclusion criteria).

Supplementary Figure S2

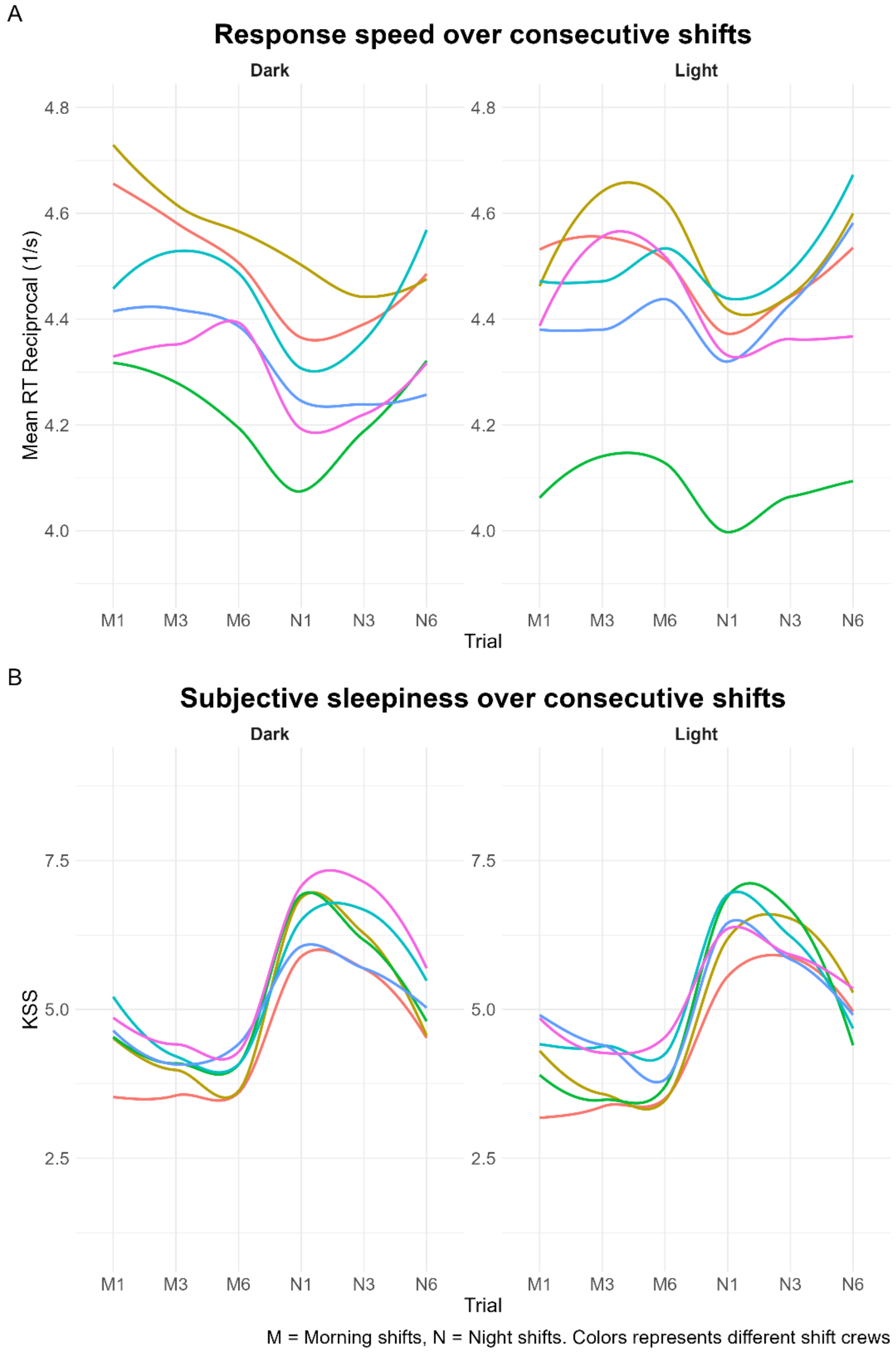

Supplementary Figure S2: Alertness and sleepiness across consecutive morning (M1,3,6) and night (N1,3,6). Panel **A** displays alertness as measured by the psychomotor vigilance test (PVT), quantified through response speed (inverse response time). Sleepiness, from the Karolinska Sleepiness Scale (KSS), is shown in panel **B.** Observations are grouped by shift crew and faceted by season (Left: Dark season; Right: Light season). The graph displays smoothed curves using LOESS regression to illustrate trends. Each color of the graphs represents a different shift crew, allowing for comparison both within and between the seasons.

Supplementary References

Åkerstedt, T., & Gillberg, M. (1990). Subjective and Objective Sleepiness in the Active Individual. International Journal of Neuroscience, 52(1–2), 29–37. https://doi.org/10.3109/00207459008994241

Kay, M., Rector, K., Consolvo, S., Greenstein, B., Wobbrock, J. O., Watson, N. F., & Kientz, J. A. (2013). PVT-touch: Adapting a reaction time test for touchscreen devices. Proceedings of the 2013 7th International Conference on Pervasive Computing Technologies for Healthcare and Workshops, PervasiveHealth 2013, 248–251. https://doi.org/10.4108/ICST.PERVASIVEHEALTH.2013.252078

Merkus, S. L., Holte, K. A., Huysmans, M. A., Van De Ven, P. M., Van Mechelen, W., & Van Der Beek, A. J. (2015). Self-Reported Recovery from 2-Week 12-Hour Shift Work Schedules: A 14-Day Follow-Up. *Safety and Health at Work*, *6*(3), 240–248. https://doi.org/10.1016/j.shaw.2015.07.003

Pallesen, S., Bjorvatn, B., Nordhus, I. H., Sivertsen, B., Hjørnevik, M., & Morin, C. M. (2008). A new scale for measuring insomnia: The Bergen Insomnia Scale. Perceptual and Motor Skills, 107(3), 691–706. https://doi.org/10.2466/PMS.107.3.691-706

Sparks, A. (2018). nasapower: A NASA POWER Global Meteorology, Surface Solar Energy and Climatology Data Client for R. Journal of Open Source Software, 3(30), 1035. https://doi.org/10.21105/joss.01035

Van Hooff, M. L. M., Geurts, S. A. E., Kompier, M. A. J., & Taris, T. W. (2007). "How Fatigued Do You Currently Feel?" Convergent and Discriminant Validity of a Single-Item Fatigue Measure. *Journal of Occupational Health*, *49*(3), 224–234. https://doi.org/10.1539/joh.49.224
